# The Hospital Nursing Supplement

**Published:** 1893-04-29

**Authors:** 


### "The Hospital" Nursing Mirror.

Being the Extra Nursing Supplement of "The Hospital" Newspaper.

[Contributions for this Supplement should be addressed to the Editor, The Hospital, 428, Strand, London, W.C., and should have the word "Nursing" plainly written in left-hand top corner of the envelope.]

### News from the Nursing World.

### THE NURSE DOLLS.

The Nurse Dolls have accepted an invitation to the exhibition which is to be held at the Mansion, Victoria Park, Keighley, next June, and they are asked to Blackpool in September. As the proceeds of the former are to go to the Cottage Hospital, it is specially appropriate for the dolls to give their services on the occasion. The Blackpool and Keighley Committees have arranged to re-dress and renovate them. How many of the correspondents who made such kind offers lately to help with the dolls, will assist us instead to add to The Hospital Collection of nursing uniforms? We want a sample of a pretty and suitable Cottage Hospital outfit, and we shall be glad to hear from any readers who are willing to send dolls dressed in the following uniforms, as we wish to have the collection made as complete as possible: General and Queen's Hospitals, Birmingham; Mater Misercordiæ Hospital and Rotunda Hospital, Dublin; Westminster Hospital, Royal Free Hospital, Liverpool Royal Infirmary and Mill Road Infirmary, Bedford Hospital, Berrywood Asylum, Gloucester Infirmary, Taunton Hospital, Lancaster Infirmary, New Hospital for Women, Women's Hospital, Soho Square; British Home for Incurables, and a "Queen's Nurse" with badge.

### MODERATE AND IMMODERATE EXERCISE.

Everyone who knows anything about hospital nurses is aware of their tendency to shirk daily exercise in the open air. Common sense tells them that they ought to go out, but their inclination is to stay in and rest their tired feet. They have "sufficient walking about in the wards, and plenty of active exercise there," they say, in extenuation. And although no one with personal experience of nursing can gainsay this, all intelligent women know that spirits, tempers, and eventually health, must suffer if the claims of fresh air be too long neglected. Refreshment of mind is unconsciously secured by those who, however unwillingly, take regular outdoor exercise on principle. District nurses, on the other hand, often get a great deal more walking than is at all beneficial even for healthy women, although we occasionally hear of tramcar companies, and proprietors of other vehicles kindly conveying local nurses free of charge. It certainly cannot be right for any skilled worker, such as a Queen's Nurse, to have to walk such long distances that she necessarily arrives at her patient's quite heated and exhausted. She needs to have all her powers at their best, and it is false economy to over-work or over-walk a nurse. It usually ends in her breaking down and a substitute having to be engaged. Only then is it realised that one district nurse has been doing the work which could have kept two employed, and some method of transit has at last to be arranged for, or a second permanent nurse becomes necessary.

### NOVEL SCHOLARSHIPS.

Under the heading "Scholarships for Trained Nurses," the Dorset Health Association announces that four nursing scholarships are to be competed for in May. Encouragement and cultivation of local talent is specially aimed at, and doubtless the Society will arrange that thoroughly good instruction shall be secured for the successful competitors.

### NURSES AS SCRUBBERS.

There seems to exist considerable misapprehension as to nurses' duties at the Manchester Royal Infirmary. The assertions made in a letter which appeared in the *Manchester Courier* of the 19th inst. are serious ones, for they include a statement that the nurses and probationers are required "to scour the floors of the wards . . . instead of attending to the patients," and that, in place of receiving systematic instruction, they are "left to pick up such knowledge as they can promiscuously." It must be unreliable and uncertain knowledge at best, if it has to be gained in the intervals of scrubbing floors! Of the writer's other "grievances" there are several which appear to require investigation and redress. We lately alluded in our columns to women translated from "wash-tub to ward," and from milk-cart to nursing duties; but at Manchester it is doubtful whether the word "scrubbers" or "nurses" is most descriptive of the workers in the infirmary. Which of our readers would like to be tended during a long, helpless illness by the hands of women who scour floors and patients alternately?

### QUEEN'S NURSES AT RYDE.

The Rev. A. L. B. Peile, President of the Queen Victoria Jubilee Institute, addressed a public meeting at Ryde on 15th inst. Mr. Peile spoke of his having spent twenty-seven years of his life in the island, which naturally gave him a special interest in the affiliation of the Ryde District Nursing Association with the Institute. He mentioned the pains which were now taken to ensure the efficient training of "Queen's Nurses," their hospital experience being always supplemented by six months' instruction in district work amongst the sick poor. The tone of the meeting was most cordial throughout, and there seemed every prospect of the Institute being well represented in the Isle of Wight.

### TRAINING FOR VILLAGE NURSES.

The Committee of the Gloucester Infirmary have decided, with great liberality, to give free training to women of good character who wish to become village nurses. A succession of these probationers, one only at a time, will be admitted for three months' training each. Although the period seems a short one to fit the candidates for their important and responsible future work, we feel sure that the time will be most profitably spent in the Infirmary, for the Matron is herself an experienced and highly-trained nurse, and the instruction given to the nurses is excellent. We hope other infirmaries will see their way to follow the example set at Gloucester.

### UNWELCOME RETRENCHMENT.

Additions to the staff of a district nursing association are pleasant indications, not only of more work, but also of increasing local support and interest. To nurse the sick poor in their own homes systematically and skilfully is to give ideal help to a patient and his family. When one district has had the services of a trained nurse for a season, it is quite usual for adjoining districts to desire equal advantages. It is, indeed, happily rare for those poor people habituated to the ministrations of a nurse, to be entirely deprived of this comfort. At Bristol the income of the District Nursing Association has proved so inadequate to the demands made upon it, that the committee have had reluctantly to give up one nurse. Unless steps are speedily taken to improve the financial position, it seems likely that other workers will have to be withdrawn. It is, however, unlikely that the inhabitants of such a city as Bristol will allow so valuable an institution to cut down its staff. All the workers are sorely needed by the poor, and surely funds will be soon forthcoming to maintain the association on its present basis.

### A NEW NURSES' HOME.

It is intended to open the new Nurses' Home at the Blackburn and East Lancaster Infirmary at Whitsuntide. It contains accommodation for thirty nurses, who will there be provided with comforts which were quite unattainable in the old buildings. The new home is a convenient and handsome one, and is to be furnished throughout with oak, by the generosity of Mr. and Mrs. Henry Harrison, who have liberally volunteered this handsome donation to the infirmary.

### TWENTY-EIGHT YEARS AFTER.

The Guardians of the Cheltenham Union can hardly avoid making a change in their nursing arrangements, for the present system has now been condemned by the Local Government Board, the Medical Officer of the workhouse, and the Medical Committee. In 1865 the Central Poor Law Authority strongly recommended guardians to appoint paid nurses, and to exclude pauper assistance in workhouse infirmaries. In 1893 it is a national disgrace that many infirmaries are but little better nursed than they were twenty-eight years ago. Better late than never, is all that can be said of the movement now slowly taking place. Every week some instance gets into the papers showing either entire absence of trained nursing or else such an inadequate supply of workers that the condition of the patients is but little improved by the nominal attendance which they get. Some infirmaries are constantly advertising for nurses, and they get rather avoided in consequence. Probably, however, it is from no fault of matron or medical officer that these frequent changes take place on the staff. The better these two officials understand their own duties, the more clearly can they comprehend as well as deplore the resignation of nurse after nurse who retires discomfited from work which is beyond the power of any conscientious woman to perform. An insufficient nursing staff is after all but very little better than an untrained one.

### STIRLING.

The second annual report of the Stirling Nursing Association was recently laid before the subscribers, and appeared very satisfactory as regarded the amount of work done. It is a pity that funds are not yet forthcoming to support a second nurse, but, doubtless, it will not be long ere this urgent need is supplied. It is to be regretted that such long distances have had to be accomplished on foot, in all weathers, by Miss Reay, the district nurse. Everyone at the meeting seemed impressed on hearing the amount of work which a single nurse has performed. Universal testimony was offered to her efficiency and kindness. It is to be hoped that Nurse Reay will speedily recover from the serious illness which has lately disabled this valuable worker.

### DUBLIN.

Irish nurses are likely to see something of cholera if the quarantine regulations in Dublin are not made stricter. Vessels arrive constantly from Hamburg and other towns where there was cholera last year, and we learn that so far no special precautions are taken with regard to these ships.

### THE DISABLED NURSE.

Nurse Pullan has collected 11s., and another nurse sends 10s. from Miss L. and Miss M. Peake, with a kindly note. This makes total received £13 15s. 6d., of which £5 17s. 6d. has been paid to our Disabled Nurse.

### MISAPPLIED ECONOMY.

After twenty-three years' faithful service in the Dundalk Union, Mrs. Mooney, a certified nurse, finds her annual salary is to be £5 less than she has hitherto received. She has been, she writes, "an officer of the Local Government Board" for all these years, and now suddenly finds her wages thus seriously reduced. Not because she is worth less to her employers, who own that she has always given them satisfaction, but because a fresh arrangement has been made with the nuns. Mrs. Mooney's duties have been altered but evidently not lessened, for the charge of the infants in No. 3 Ward has been given to her. We trust the guardians will consider poor Mrs. Mooney's humble request that her salary "should remain as heretofore." Truly, it is discouraging to all classes of workers to see long and faithful service acknowledged only by reduced remuneration.

### MORE NURSES FOR INDIA.

The words "Europeans in India" are of personal interest to most of our readers. Everybody has either friends or acquaintances in that great empire, and therefore all must be glad to hear of a practical scheme for benefiting these residents. The haze of romance through which India is pictured by the untravelled European, is oftimes rudely dispelled by accounts of acute illnesses running a rapid course, reducing the strength of the men, and making the women into chronic invalids, because skilled nursing cannot be secured for them at critical times. In another column our readers will find an account of the new Nursing Association, but they must bear in mind that it is still in its infancy. Much has to be accomplished ere any substantial augmentation of the present nursing staffs in India can be hoped for. However, we must all wish that success will attend this excellent new departure, and that the Committee will prove right in its modest estimate of the cost of supplying these additional nurses.

### SHORT ITEMS.

The nucleus of a good nurses' library is already laid at the Mater Misercordiæ Hospital in Dublin. Evidently the nuns intend the training of their probationers to be efficiently provided for.—The council of the Newport (I. of W.) Nursing Society is appealing for subscriptions to cover the cost of another nurse.—Miss Musgrove, of the Manchester School of Domestic Economy, is delivering a course of lectures on "Sick Nursing" at Levens.—At a public meeting at Wick the other day, it was unanimously decided to form a "Wick and Pulteneytown District Nursing Association," and to seek affiliation with the Q. V. J. I.—Under the presidency of the Marchioness of Londonderry, a drawing room meeting was recently held at Comber, at which it was agreed to establish a trained district nurse in the town.

### Sanitation for Nurses.

By a Sanitary Inspector.

### III.—NOTIFICATION IN FOREIGN COUNTRIES.

When we come to consider the state of sanitation in Belgium, we are at once struck with the perfection to which the science has attained in that country, and this is the more strange because Belgium has had no recent Public Health legislation, but the regulations are based only on an old French law, dating back as far as 1794, and still in force in the main, although some statutes have been added recently to bring it up to modern requirements.

In Belgium the supreme sanitary authority is either the Burgomaster or the Bureau d'Hygiène, and to one or the other of these authorities all doctors have to notify every case of infectious disease which occurs in their districts. In the case of asylums, prisons, hotels, lodging-houses, inns, &c., the heads of these institutions have to notify the outbreak of infectious disease.

The notifiable diseases in Belgium are small-pox, scarlet fever, typhoid, cholera, measles, typhus, dysentery, and diphtheria.

In the country there has been founded a Provincial Medical Commission for each separate province, and this body is responsible for all health matters, and the sanitary welfare of its special province. In 1880 a statute was passed which laid down in great detail the duties of this Provincial Commission. Everything which affects the public health is the concern of this body, which has also to report all defects in, or non-compliance with, the sanitary regulations which have been laid down for their guidance. When any infectious disease breaks out in a province, the president of its Provincial Medical Commission is obliged personally to visit the locality, and to decide what precautions ought to be taken, and he has then to consult with the local authorities as to the means by which the measures decided on by the president shall be carried out. Finally, it remains still for the president to report to headquarters, viz., the Bureau d'Hygiène, the nature of the disease and the precautions which he has thought right to enforce. The Bureau d'Hygiène in Belgium is an extremely well-organised and efficient one. The sanitary regulations constitute a somewhat cumbersome piece of machinery in Belgium on account of the great minutiæ of the details, the number of various officials to whom application has to be made, and the authorities to whom matters have to be ultimately referred. In the Burgomaster is vested the sole right, without appeal, to have insanitary houses pulled down, or to order sanitary inspection of private houses. Thus the Burgomaster possesses very considerable power, and he is only responsible to the Minister of Agriculture, Industry, and Public Works, since he adds the supreme control over sanitary matters, in this country, to the other duties of his office.

In Brussels the regulations for infectious diseases, date back as far as 1824, and it speaks volumes for the completeness of these, that they are still in force. It is there decreed that any doctor as soon as a case of infectious disease comes to his notice shall report it to the Burgomaster, and special certificates are drawn up for the purpose, with which every doctor is provided. Afterwards it is the duty of the Officer of Health to take up the matter, and make a thorough inspection of the sanitary arrangements in the patient's house, and the usual inquiries into the probable cause of the disease originally.

Hitherto no town in Belgium, not even the capital, provides special hospitals for infectious diseases, but such patients are nursed in special pavilions connected with the general hospitals and isolated as far as is possible. Even small-pox has not a special hospital provided, but is nursed on the top storey of the general hospital. Typhoid, as in England, is nursed in the general wards.

The regulations for children attending school when infectious disease breaks out, are the same as those which hold in England.

In a recent work Dr. Palmberg gives an interesting description of the simple but very ingenious plan adopted by Dr. Jannsen, the Director of the Bureau d'Hygiène, for keeping a clear record of infectious diseases. He has a large and elaborate plan of the town, and uses pins of various colours which he sticks over the houses where infectious disease has appeared. Thus for typhus he uses yellow pins, for small-pox black ones, for scarlet fever red ones, and so on. Moreover, he further distinguishes new cases from old ones by only inserting half-sized pins to indicate the former.

Thus do sanitary matters stand in Belgium. We will now turn to Austria. Here the State is responsible for the proper superintendence of hospitals, and for supervision of infectious diseases, epidemics and quarantine; the head authority regarding public hygiene being the Minister of the Interior, all matters being referred to him by the chief medical officer called the "Sanitatsouferent," who is always a qualified medical man. If the minister requires the advice of experts on any point, he assembles the higher Sanitary Council composed of at least six members, all doctors of eminence, and to these the matter is referred. Whilst, however, the State holds the chief authority, it makes the various communes responsible for carrying out the various precautionary measures laid down by the State against infectious diseases, and the heads of each commune have to send in full reports of proceedings.

The regulations for the prevention of infectious diseases are very good. Regulations are drawn up for the guidance of the doctors when an epidemic breaks out, and amongst other things they are required to "make a summary of the former reports, show the result of the measures employed, and propose improvements for the future," which shows a praiseworthy desire on the part of the authorities to investigate epidemics, even if they cannot stamp them out. Another regulation that in case of the occurrence of a "deficiency in the number of doctors, nurses, or hospitals it must be provided for without delay," might under some circumstances, we should imagine, be very difficult to carry out.

In Vienna the chief direction of sanitary matters is entrusted to the "Medical Bureau" comprising a doctor with several assistants under him, whilst the practical part, the direction and efficient carrying out of preventive measures, devolves on the district doctor, who is to be given every facility for the performance of his duty by all the authorities of the Commune.

In Austria we find notification of infectious diseases compulsory, and the following diseases are those that have to be notified: whooping-cough, typhus, small-pox, scarlet fever, erysipelas, measles, dysentry, diphtheria, puerperal fever, purulent ophthalmia, hydrophobia, and trichinosis. It is a curious fact, that whereas such uncommon diseases as hydrophobia and trichinosis are notifiable, typhoid fever is not among the number, an almost unique exception, we should imagine, as regards continental countries, and hardly in keeping with the generally excellent precautionary measures in fever. The doctor, in whose practice the case of infectious disease occurs, has to report it to the district doctor, and is then relieved of all responsibility in the matter, as the district doctor has to decide on all precautionary measures, also has to see to the removal of the patient to hospital, and to the disinfection of the house, and grants a certificate that all precautions have been adopted and disinfection efficiently carried out.

In no part of Austria—not even in Vienna—are there any special hospitals for isolating infectious dieases, but the usual continental plan is adopted of special pavilions to the general hospitals for the nursing of infectious diseases, and where naturally isolation can only be very imperfectly carried out.

### A German Hospital.

By an English Patient.

Having gone to Germany merely to study the language, I found myself, in the course of a few weeks, established as a patient in the scarlet fever ward of a hospital. Never having previously entered such an institution, everything was quite new and strange to me, apart from the fact of it being a hospital in a foreign country.

On my arrival I was eaten up with misery, and lay under the cover and wept, and wished myself at home—quite like a German girl, in fact. That fair creature is exceedingly tender, and easily moved to tears upon all occasions, whether of joy or sorrow—at least, that is true of the girls of the lower classes such as surrounded me there.

The hospital, which is comparatively new, is considered a very fine one, and is out in the country, some two miles or more from a town. The extensive grounds are well laid out, planted with trees and flowering shrubs, which looked lovely in the middle of May. It was quite amusing to look from any of the windows: groups of male convalescents, dressed from head to foot in white, standing at corners smoking their pipes; girls in grey speckled gowns walking slowly up and down, knitting, or sitting on benches in the sun; nurses always flitting about, and here and there a doctor—easily recognised by his coat—scudding along, closely followed by an attendant "over-nurse." The wards, or pavilions, are 55 in number, and of various sizes, dotted all about, each one being entirely separate from the others, consequently there is an abundance of light and air in each ward.

I will endeavour to give The Hospital readers some idea of my own personal experiences and observations in one particular ward, as there is only one class, viz., the third, for scarlet fever patients.

The ward is divided into three parts; the middle, and by far the largest part, being the actual ward itself. At one end is the "day-room," divided from the ward by sliding doors," which, however, are always open. It is furnished with two large tables, wooden chairs, and benches. The wall on one side is composed of nothing but windows, and during the hot weather there is an awning outside. The wall dividing the day-room from the ward is also all windows, so the nurse can sit still and yet see all that goes on.

The nurses and convalescents take their meals in the day-room, and the latter are supposed to sit there during the day. From this room there is an exit into the garden. In a corresponding position to the day-room, and also communicating with the ward by sliding doors, are two rooms at the other end of the ward; these are a large bath-room, and a small bed-room for the head nurse. Between these two rooms is a passage, at the end of which is a door leading into the garden.

There are nineteen beds in this ward, and at the side of each there stands a little table. In a line down the middle of the room are a writing table, a case containing medicines and various appliances, the heating apparatus, and a marble table. I should have said, in speaking of the beds, that over each one hangs a little blackboard, on which the nurse, by means of numbers and coloured chalks, silently conveys to the doctor every possible information regarding the patient. It is a very complicated arrangement, and has to be explained to every new doctor.

The day seems very long, for it begins at six a.m., when we all with one accord awake, owing possibly to the current of fresh air. The nurses and convalescents rise and the children begin to make a noise, so there is no chance of more sleep for anybody. At half-past six bread and butter and coffee are brought round—such coffee! Never in my life before had I drunk such a wicked mixture under the name of coffee! This is the more astonishing because the Teuton knows what good coffee is, and apparently could not live without it. However, I actually grew accustomed to and even looked forward to it in time. After many complaints my portion had the virtue of heat, which, in my opinion, partially covered the other defects.

All the food becomes more or less cold owing to the fact that there is one kitchen for the entire establishment, and it is five minutes' walk from the ward I am describing, and consequently the food cools during transit. There is a kitchen, capable of containing two people comfortably, attached to each ward, but the only cooking convenience is a gas jet, over which the nurses can make things hot for people who, like me, largely indulge in the Englishman's privilege of grumbling.

Imagine my consternation on hearing that bread and butter are not provided for any patients except children, in a third class ward, so each has to supply herself.

After coffee the nurses and convalescents are busy sweeping, mopping, and drying the pretty tiled floor of the ward. The head nurse was evidently very proud of her ward, and thought nothing of polishing for an hour or more at one small glass case. She cleaned all the windows on one occasion, and to my amazement opened each quite wide in order to clean it outside, utterly regardless of the patients whose beds stand against the glass. My window she did not open, for I objected so strongly.

When the ward had arrived at the requisite degree of perfection, it was our turn to be beautified.

About nine o'clock the first supply of milk came in, and the second at six in the evening. The milk is of two kinds, blue milk for those over 14, and new for the children. Each patient receives rather more than half a-pint, it having been previously measured in the kitchen, so that there is rarely more than enough when the nurse measures it again in the ward for the patients. Breakfast, about ten o'clock, consists of an egg, smoked or salt fish, or sausage—the meat, of whatever kind, having also been measured exactly in the great kitchen—bread and butter (supplied by the patients), and sometimes a kind of thin soup flavoured with fowl, but I think they make about a gallon out of one chicken. I learnt to use my hand as an egg cup, a feat which requires some little dexterity, but there were no others to be had in the Third Class Ward. All the food is served in enamelled metal vessels, which would not be so objectionable if the enamel did not break away, leaving bare the black metal, and it is not unusual to find loose pieces lying at the bottom.

(*To be continued.*)

### Notes and Queries.

### Queries.

(115) *Training.*—I shall be very glad of advice as to how to set about getting trained as a hospital nurse?—*Nellie.*

(116) *Massage.*—I have no hospital experience and no knowledge of anatomy. Can I learn massage without these? and how soon should I be in a position to earn anything?—*Subscriber.*

(117) *Mental.*—I am anxious to be trained as nurse or attendant on mental cases, and shall be glad of advice as to how to begin?—*A. H.*

(118) *Spinal Caries.*—Please tell me in which numbers of The Hospital articles on this subject recently appeared, also where can I get the back numbers?—*Sister Mary.*

### Answers.

(115) *Training* (*Nellie*).—We very often reply to this question in our "Notes and Queries." Read back numbers of The Hospital, and get "How to Become a Nurse," by Honnor Morten; published by Scientific Press, 428, Strand.

(116) *Massage* (*Subscriber*).—You had better get terms, &c., from any well-known teacher. Your being likely to make a living by massage must depend on your own aptitude, and also on your having thoroughly good instruction. You wil find advertisements in our columns.

(117) *Mental* (*A. H.*).—Write to the medical superintendent, Berry Wood, Northampton.

(118) *Spinal Caries* (*Sister Mary*).—You can get back numbers of The Hospital at the offices, 428, Strand. The article you ask about is in February 11th, 1893.

### The Jubilee Institute at Taunton.

Mr. H. R. Sears, the Chairman of the Taunton and Somerset Hospital Committee, writes that the statement made in these columns on the 22nd inst. "is absolutely untrue in every particular." We pointed out that this institute was founded to provide nursing in their own homes for the sick poor of the town and district. We presume this is the statement referred to, and its accuracy may be gathered from the following summary of a discussion which has taken place in the *Somerset County Gazette*. This paragraph appeared in that paper earlier in the month: "The nursing question in Taunton is not likely to be dropped. The attitude adopted by the Taunton District Nursing Association, upon the initiative of Mr. Cosens, is too significant and important to be overlooked. A reference to our correspondence columns to day will afford indication of the frame of mind in which the subject is regarded. The provision of nurses for the sick poor was one of the avowed objects for which an appeal was made for funds in aid of the Jubilee Nursing Institute. That institute is doing its work in the homes of the well-to-do exclusively; for, as Mr. Cosens declared, 'I can safely say that the institute has never lent a nurse for the sick poor in the whole of Taunton.' The money, 'a large sum,' subscribed towards the Jubilee Institute upon 'the understanding that nurses should be provided for the poor' has never yet been applied to that purpose. This is a grave state of affairs, and there is no getting away from the conclusion it drives home. Reparation can only be made in the way suggested by Mr. Cosens and approved by the meeting which he addressed, and we trust, therefore, that the Committee of the Taunton Hospital will, as soon as possible, and without a dissentient voice, make a handsome grant in aid of the funds of the Taunton District Nursing Association, which is courageously striving to carry out some of that philanthropic work which the institute has all along neglected. The case as between the Nursing Association and the Hospital Committee is not one of condescension or concession, much less one of charity. The position is this: Funds have been subscribed towards the Jubilee Nursing Institute in order that its nurses should render certain services which they do not render, and which the establishment has never rendered. The District Nursing Association upon the other hand, with limited means, is bravely endeavouring to perform those offices for which the funds of the Taunton Jubilee Nursing Institute were augmented. The local demands for aid altogether exceed the resources of the Nursing Association. In the interests of humanity, this excellent agency deserves to be liberally assisted. At least it should get back its own money." In the same paper a correspondent, in a letter too long to quote entirely, says: "It seems to me that the remarks made by Mr. Cosens at the annual meeting of the Taunton District Nursing Association held last week, represent a feeling which has long existed in the minds of many Tauntonians in reference to the Jubilee Nursing Institute and the object with which it was founded, viz., the provision of nurses for the sick poor in the town and the whole of the surrounding district." Altogether, the general view taken of the original object of the Jubilee Nursing Institute at Taunton seems to be the same as that which was given in a paragraph which appeared in The Hospital of April 22nd.

As the *Somerset County Gazette* of the same date contains a letter from the Chairman of the Taunton and Somerset Hospital Committee on this subject we will give it in full:—

"You will find a full account of the public meeting *re* Victoria Jubilee Nursing Institute, in the *Somerset County Gazette* of January 15th, 1887. The object is there stated in one of the principal resolutions to be "the training and accommodation of nurses for the hospital and general public," and nowhere, I think, will you find in the whole proceedings any mention of a promise to provide for gratuitous nursing of the poor outside the hospital. Therefore, the statement that the hospital or institute authorities have betrayed a trust or broken a pledge is untrue, and calculated to do great mischief.—Yours faithfully,

"R. H. Sears.

"Priory, Taunton, April 20th, 1893."

There is also a short letter from Mr. Cosens in the same issue of the *Somerset County Gazette*: "Sir,—As I am answerable for the request from the above Association that the Nursing Institute should make a grant to them out of their funds, on the plea that moneys were subscribed to the Jubilee Fund for the express purpose of nursing the sick poor of the town, may I be allowed to state that the impression that I and many others entertained as to the use of the fund was erroneous? I can find no mention of nursing the sick poor out of the hospital as part of the scheme laid down at the great meeting held in the Shire Hall. With these few words, may I ask the kind support of your readers for the Taunton District Nursing Association?—Yours faithfully, "W. Burrough Cosens."

With reference to the foregoing letters, the *Somerset County Gazette* points out, (1) that the position taken by it prior to the annual meeting of the Taunton District Nursing Association, held on March 21st, was one of regret only as to the absence from Taunton of any agency for supplying free nursing to the poor in their own homes, excepting only the recently-formed District Nursing Association—an excellent organization certainly, but sadly limited in point of resources. (2) That the observations made by us as to the obligation of the hospital authorities towards the work of the District Nursing Association were based upon a resolution, moved by Mr. Cozens, honorary surgeon of the hospital, seconded by Mr. Chapman, ex-Mayor of Taunton, and unanimously carried at a meeting attended by governors of the hospital, declaring that "The chairman be requested to ask the committee of the Taunton Hospital to make a grant to the funds of the association, seeing that a large amount of money was subscribed to the Nursing Institute on the understanding that it would be for the nursing of the sick poor." (3) That the honorary surgeon of the hospital affirmed, without contradiction, at the meeting referred to, that "when the Jubilee Nursing Institute was started some years ago, and an appeal was made for funds, one of its objects was stated to be the provision of nurses for the sick poor, but he could safely say that the institute had never lent a nurse for the sick poor in the whole of Taunton." (4) That the words of the resolution quoted by Mr. Sears from the proceedings of the county meeting held at Taunton on January 8th, 1887—namely, "That the sum to be so raised shall be applied to the development of the Taunton and Somerset Hospital, especially in providing and maintaining a building which shall be called 'The Victoria Jubilee Nursing Institute,' and which shall have as its object the training and accommodation of nurses for the hospital and the general public"—do not, to say the least, prove an absence of intention to nurse the sick poor, who are unfortunately part of "the general public"; and (5) those who are in favour of a grant in aid being made to the cause of such nursing as represented by the Taunton District Nursing Association will be gratified to learn that the appeal for assistance, which might have been at once refused, or even ignored, is, as a matter of fact, being considered by a sub-committee of the hospital.

### HIDDEN DANGERS.

We read that two navvies suffering from small-pox in Derbyshire were conveyed in a cart used to take round bread. The distribution of "the staff of life" was calmly continued after the occurrence of this untoward incident.

### Nursing Subjects at Chicago.

We print from *The Trained Nurse* of April, the accompanying list of the papers which are to be read and discussed at the Congress. The selection is certainly admirable and includes all important subjects connected with hospitals, asylums nurses, and training schools. Miss Nightingale's paper is very properly placed at the head of the sub-section, and we know that her name is as well-known and as honoured by American nurses as by their English sisters.

"Quite a general interest has been expressed in the section on hospital care of the sick, training of nurses, &c., in the World's Congress Auxiliary at the World's Fair, and the outlook is very encouraging for much profitable interchange of thought. Let us hope that, in addition to the regular Congress, something will be done towards forming a Superintendent Convention, and through it work out a general American Nurses' Association, which never will come until the Superintendents of schools have ideas and feelings in common.

Through the kindness of Miss Hampton, of Johns Hopkins Hospital, we are able to present the following list of papers which have been promised for the Congress:

1. "Hospital Accounts and Methods of Book-keeping." By Mr. James R. Lathrop, Superintendent Roosevelt Hospital, New York.
2. "The Church Hospital." By A. Rittenhouse, D.D., Superintendent Methodist Episcopal Hospital Philadelphia.
3. "Infectious Wards in General Hospitals." By Dr. G. H. M. Rowe, Superintendent of The City Hospital, Boston.
4. "Detention Hospitals for Insane and Alcoholic Cases.' By Dr. Matthew D. Field, Examiner in Lunacy, New York.
5. "Special Conditions of Organisation Presented by American Hospitals." By Dr. L. S. Pilcher, Brooklyn, N. Y.
6. "Hospital Saturday and Sunday." By Frederick F. Cook, General Agent Hospital Saturday and Sunday Association, New York.
7. "Diet Kitchens in Hospitals." By Dr. H. B. Stehman, Superintendent Presbyterian Hospital, Chicago.
8. "The Relations of the Medical Staff to the Governing Bodies in Hospitals." By Dr. Edward Cowles, McLean Asylum, Somerville, Mass.
9. Dr. Clarence J. H. Chipman, House Surgeon, General Protestant Hospital, Ottawa, Ont., will discuss "Hospitals, their Construction and Management."
10. "Obstetric Hospitals." By Dr. B. C. Hirst, University of Pennsylvania Hospital, Philadelphia.
11. "Hospital Plans for Contagious and Infectious Diseases." By Dr. M. L. Davis, Lancaster, Pa.
12. Dr. Samuel Bell, Member of Board of Charities and Correction of Michigan, will discuss "Relations of Hospital to Increase of Knowledge."
13. "Hospital Laundries and Means of Disinfection." By Dr. A. C. Abbott, University of Pennsylvania, Philadelphia.
14. "Naval Hospitals." By a Surgeon in the Navy.
15. "Paying Patients in Hospitals." By Dr. Henry M. Lyman, Attending Physician Presbyterian Hospital, Chicago, Ill.
16. "The Organisation of Boards of Trustees of Hospitals and their Duties." By Richard Wood, President Board of Managers of University of Pennsylvania Hospital, Philadelphia.
17. "An Account of the Hospitals of Chicago." By Geo. B. Dresher, Superintendent St. Luke's Hospital, Chicago.
18. "An Account of the Hospitals of Boston." By Dr. G. H. M. Rowe, Superintendent of the City Hospital, Boston.
19. "The Royal Victoria Hospital of Montreal." By John Robson, Secretary Royal Victoria Hospital, Montreal.
20. "The Hospitals of New York and Brooklyn." By Dr. L. S. Pilcher, Methodist Episcopal Hospital, Brooklyn, N.Y.
21. "Relations of Training-Schools [to] Hospitals." By Miss Dock, Johns Hopkins Hospital.
22. "Nurses' Homes." By Miss Lett, St. Luke's Hospital, Chicago.
23. "Laundries." By Miss Kimber, New York City Training-School.
24. "Educational Standards for Nurses." By Miss I. Hampton, Johns Hopkins Hospital.

The Following Papers are Promised from Abroad.

25. "The Ethics and Principles of Hospital Construction and Management." By F. J. Mouat, London, England.
26. Paper not stated. B. W. Richardson, London, England.
27. "On the Utility, Peculiarities, and Special Needs of Hospitals for Children." By William Wallis Ord, London, England.
28. "Cottage Hospitals." By Francis Vacher.
29. "First Aid to the Injured in Peace and War." By John Furley.
30. Paper (French) Nursing). L. N. Worthington, Paris, France.
31. "Systems of Hospital Nursing in Amsterdam." By T. Edward Stumpff.
32. "Paper (subject to follow). By A. Pearce Gould, London, England.
33. "Hospital Finances." By Henry C. Burdett, London, England.
34. Paper (subject to follow). Lord Cathcart.

American Subjects.

1. "Training Schools in America." By Miss I. Sutliffe, New York Hospital, New York.
2. "Proper Organisation of Training Schools in America." By Miss L. Darche, New York City Training School.
3. "Alumni Associations for Nurses." By Miss I. Merritt, Brooklyn City Hospital.
4. "Private Duty Nursing." By Miss Hintze, Boston City Directory of Nurses.
5. "Nurses as Heads of Hospitals." By Miss L. Davis, University Hospital, Philadelphia.
6. "Nursing of the Insane." By Miss May, Rochester State Hospital, Rochester, N. Y.
7. Miss A. Maxwell. Presbyterian Hospital, New York, will discuss "Training Schools in America."
8. Miss Annette M. Summer, Editor *Trained Nurse Magazine*, New York, will discuss "Proper Organization of Training Schools in America."
9. Miss MacIsaac, Assistant Superintendent Illinois Training School, Chicago, will discuss "Alumni Associations for Nurses."
10. Miss Pope, Columbia Hospital, Washington, will discuss "Obstetric Nursing."
11. Miss Drown, Boston City Hospital, will discuss "Nursing of Infectious Diseases."

Sub-Section on Nursing.—Papers Promised from Abroad.

1. "The Principles of Nurse Training." By Miss Florence Nightingale, London.
2. "British Nurses' Association." By the Princess Christian.
3. "Nursing in Scotland." By Miss R. F. Lumsden, Aberdeen Royal Infirmary.
4. "Workhouse Nurses' Association." By Miss L. Twining, London.
5. "Infirmary Nursing." By Miss Josephine De Pledge, Chelsea Infirmary, London.
6. "District Nursing." By Mrs. Dacre Craven, London.
7. "Trained Nursing in Italy." By Miss A. S. Martin, St. Paul's House for Nurses, Rome, Italy.
8. "Nursing in Almshouses." By Miss A. C. Gibson, Birmingham Workhouse Infirmary.
9. "Nursing in Sanatoriums and Home Hospitals." By Mrs. Bedford Fenwick, London.

### The Vaterlandische Frauenverein.

This is the title of the Society for the Care of the Sick and Wounded, which held its annual meeting on April 14th, at Berlin. The Empress of Germany presided on the occasion. The Society numbers 111,000 members, in all parts of Germany, and much valuable work was done by it during the cholera epidemic of last year, trained nurses being supplied to Hamburg, &c. The Society also gave up a hospital of its own for the special use of cholera patients. It is said that over 600 trained nurses and 570 assistant nurses could be provided for service at any moment, if war broke out, and that provision is also made for temporary hospitals, ambulances, and convalescent stations. Home nursing, hospitals, and the management of nurse training institutions engage the attention of the Society in times of peace, and it seems to undertake a vast amount of work.

### Up-Country Nursing Association for Europeans in India.

This recently-founded association designs to supply trained nurses to far-away Indian stations—where they are indeed urgently required. Not by starting any new and costly organisation is this to be done, but by enabling existing institutions to add to the number of their workers. These supplementary nurses are to be available for the out-of-the-way districts already alluded to, which are at present entirely unprovided for. Many Europeans are quite unable to obtain nurses, however serious their illness. They cannot afford to pay the costly travelling expenses in addition to the fee, which is about £10 a month. Even if they could do this, their getting a nurse sent up-country to them is a difficulty not easy to overcome, for the supply of English nurses now in India is quite inadequate to meet the demand. Ten times as many are needed to do the work which is waiting for them. Subscriptions are asked for to cover the initial expenses of the association. The committee estimate that £50 will suffice to export and establish one nurse in India. In other words, to place each one "within a reasonable distance at a reasonable cost" of future patients. H.R.H. the Duchess of Connaught has expressed warm sympathy in the scheme, of which the Hon. Mrs. Neville Lyttelton, 21, Carlton House Terrace, is the Hon. Treasurer; Major-General J. Bonus, R.E., The Cedars, Strawberry Hill, the Hon. Secretary; and Lord Brassey the President. The Vice-presidents are the Duchess of Sutherland, Countess Cowper, Viscountess Galway, Lady Herschell, Lady Lyall, Sir Arthur Hamilton Gordon, Sir James Peile, Sir James Lyall, Sir Frank Forbes Adam, Sir H. Seymour King, M.P., Mr. Henry Gladstone, and Mr. Pandeli Ralli.

The Working Committee contains: Countess Cowper, Lady Helen Munro Ferguson, Hon. Mrs. Neville Lyttelton, Lady Lyall (Alfred), Lady Lyall (James) Mrs. Sheppard, Mrs. Robertson, Mrs. Cuthell, Sir James Peile, Sir Frank Forbes Adam, and Surgeon-Colonel Butler Hamilton. Under such favourable auspices we can surely hope ere long to hear that every up-country station in India has a prospect of a skilled nurse within its reach.

### Appointments.

Fletcher Convalescent Home, Cromer.—Miss Harman-Brown has been appointed Matron of the New Fletcher Convalescent Home. Miss Harman-Brown was Matron of the Tunbridge Wells Ear and Eye Hospital, and has also had varied and valuable nursing experience. We wish her every success at Cromer.

Wolverhampton Borough Hospital.— Miss Annie Mulligan has been appointed Matron of the Wolverhampton Borough Hospital. Miss Mulligan was trained at Sir Patrick Dun's Hospital, Dublin, and afterwards held the post of Sister at Fleming Memorial Hospital, Newcastle-on Tyne, and at Monsall Fever Hospital, Manchester.

### Where to Go.

Miss Esther Palliser, Madame Belle Cole, Madame Clare Samuel, Mrs. Helen Trust, Madame Hope Glenn, Mr. Iver McKay, Mr. C. Chilley, Mr. Joseph O'Mara, Mr. Edwin Houghtin, and Sir Charles and Lady Hallé have promised their gratuitous services at the concert to be given by the Stock Exchange Orchestral Society on May 9th in aid of the Chelsea Hospital for Women. Tickets may be had from the Secretary, at the Hospital, in the Fulham Road.

### For Reading to the Sick.

### THE PRESCRIPTION.—II.

The prescriptions which our Heavenly Father thinks suitable for our complaints are usually very unpalatable, as they go against our natural tastes and wishes ; nevertheless we are obliged to take them, whether we will or no. We believe with our minds that "all things work together for good," but our hearts refuse to realize it in our own case. When our bodies are sick and the physician has prescribed for us, we get the medicine made up by the chemist, who takes a small quantity of one ingredient from one bottle, and some from another, and again he goes to a third, or even, perhaps, a fourth. Why he does so we cannot tell, only when the whole is properly mixed together, the result is a cure. Any one of those drugs taken separately or in a large dose might have killed us outright, but the skilful practitioner knows just the proper proportions which will benefit us. In the same way our Great Healer deals with our souls, He never sends us more of one affliction than we can endure. There are wonderful compensations mixed with the chastisements He sees necessary for His children's welfare. It is not one thing by itself, but one or two or three things together which will do us good. Sickness, which cuts us off from enjoyment, is accompanied by the extra care and sympathy of our friends, who lighten our sufferings by their loving tenderness, qualities we did not previously think they possessed. God gives us "plenteousness of tears to drink" when we sorrow for the loss of "our dearest and our best," yet by this cross we are drawn nearer to Him who is our Father indeed, for guidance and support, and tenderer than a mother towards our weaknesses and falls. Disappointments check our inordinate desire for earthly advancement, slights, whether or not merited, make us humble, and bitter medicines, though they be sour and rough to the palate, yet are an excellent tonic to strengthen us for our warfare with self and sin, and help us to find our happiness in a better and purer life.

We will not look in dark suspicion upon the blows of affliction which fall upon us, but wait in patience to see with what blessings they are combined. "Every cloud has its silver lining," says the proverb, and we shall find, sooner or later, that it is worth while to have the bitter, if it be needed, as the basis of the wine of life, so sweet to the taste.

"Ye fearful saints, fresh courage take,
The clouds ye so much dread
Are big with mercies and will break
In blessings on your head."

### Presentation.

Miss Emma Loveys has been presented with an electro-plate tea service by her nurses as a mark of their affectionate appreciation of her constant kindness to them during the two years in which she has been superintendent of the Wolverhampton branch of the Queen Victoria Jubilee Institute.

### Wants and Workers.

The Rev. Grant Mills, Hospitaller and Librarian of St. Thomas's Hospital, will be glad of contributions to the patients and nurses' libraries. It is thirteen years ago since these were first formed, and they are much in need of help at present.

"District Nurse", Pinner, Middlesex, will be much obliged for information regarding any place where a spinal carriage, suitable for a girl of ten, can be borrowed or hired for a few months. Great care will be taken of it.

The London Shoe Company, 116 and 117, New Bond Street, having been entered by burglars, and goods to a very large amount stolen, an impression has got abroad that they would be unable to supply without delay all the requirements of customers. The Company were, however, able to replace the loss within three hours from their City warehouse.

### Her Better Self.

### II.

For a spell, deeper plunges into fashionable dissipation helped to stifle troublesome thoughts. But the latent good in the country girl's character had been stirred, and there came a day when Daphne Reece actually sat down to think. On her lap lay a home-letter in the sea-captain's cramped handwriting. It was full of those scraps of country news that somehow sounded so narrow and dwindled in the atmosphere of the town. On every page Harry Dunsford's name figured frequently. He seemed to be incessantly at the bunk, smoking pipes with the old sailor and cheering his dulness. "He's quite like a son to me, in fact," wrote the old man, in the innocence of ignorance. Oddly enough, Daphne never missed a shred of news about Harry, though she told herself loftily that never again could things be as they had been between herself and him now that she had tasted life as it really ought to be lived according to her new code. Nevertheless, Daphne's awakening was dawning. One afternoon, in a Bond Street block, while Aunt Sarah, picking out, through her long-handled *pince-nez*, this celebrity and that, was reeling off spicy histories of each to pass the time until her carriage could move on, Daphne's eyes were arrested by a face in a neighbouring vehicle. It was a woman's face; the features were purely cut, and the softly-folded lips were firm in their lines; while the eyes, blue and steadfast, that looked neither to the right nor to the left of the fashionable waiting throng, were fixed watchfully on a reclining figure by her side, a lad whom the most careless onlooker could see was fading out of life swiftly. They were nurse and patient—as the modest uniform of the one told —belated in the afternoon block by some untoward accident. Daphne gazed at them, at first idly, then the sharp contrast between the earnest, purposeful face of the nurse to those others around struck her. It was on the evening of that day Daphne sat down to think. "Life was real, life was earnest," she remembered that somebody had said, and the face she had that day seen, so full of its owner's life-purpose, confirmed the fact. There was that stuff in the old sea captain's daughter that urged her, once convinced, to be likewise up and doing.

But what to do was the question that floated through her brain as she hurried on from one festivity to another with the untiring Aunt Sarah. Visit the sick? Well, that was easily done; and next morning, long before Aunt Sarah came down to luncheon, Daphne had been out to provide herself with flowers and toys, for she was bent on a visit to some children's hospital, small beginnings as she said to herself.

"Oh, Miss," tearfully piped out Masters, Aunt Sarah's maid, who accompanied her, "I'm that sorry! I do wish as you'd give it up. It'll never do. Long ago, there was a young lady, one of the mistress' excuses as she calls 'em, well, she was took serious. It's my private belief as it was along of an 'igh church curate, but howsomever, she come to a bad end, she did."

"How? What was it?" asked Daphne breathlessly. "Was she run over in the street?"

"A deal worse, Miss!" said Masters, with intense solemnity, "She's single, to this day!"

The spring had crept on apace down in the country, while Daphne danced and laughed the hours away in town. It had been a busy time for Harry Dunsford, then came a lull while Nature was let alone to do her part, and the full force of his desolation came home to him. He had not even the comfort of letters. "Only properly engaged people correspond! And we're not that!" Daphne had decided, and Harry had to be content with visits to The Bunk, where all things reminded him of the graceful presence no longer there.

"A downright good lad!" the Captain never failed to remark each evening, when Harry, after a spell of sea-yarns, took his leave; and the old man would return to his pipe and build his air castles. What a thing it would be if Daphy would but take a fancy to this upright, gentlemanlike, young fellow; come of a good stock, too, and means enough—especially when the girl's bit of money should be added—to live in a tidy fashion. It was time Daphy was back from London! The old man suddenly resolved to order his daughter home, a vow that was smoked into a doze, then into a profound sleep.

*(To be continued.)*

### Maternal Sanitation.

In these days we lay such stress on isolation for infectious cases, and talk so much about preventable diseases, that we almost believe the whole community is willing to assist in stamping out epidemics. But this pleasing delusion is dispelled when we hear of a proceeding like the one lately brought to the notice of the Whitchurch guardians. The Medical Officer of Health, reported the case of a child who had contracted scarlet fever. The mother was doubtless distressed at the occurrence and the trouble it entailed, and she thereupon appeared to decide to limit the inconvenience to a definite period. She frankly explained to the medical officer that she had put four other children to sleep in the same room with the patient to ensure their catching the fever. One child, however, succeeded in escaping the contagion, and his parent was rather resentful of his immunity. Our sanitary progress is certainly liable to receive checks which are as effectual as they are surprising.

### The Infectiousness of Consumption.

### DIRECTIONS TO PATIENTS AND THEIR FRIENDS.

The following directions to out-patients have been prepared by the medical staff of the North London Consumption Hospital.

These directions have a much wider application, and should be borne in mind not only by patients but by their friends, and all who have the nursing or care of these cases.

As it is now known that the phlegm coughed up in consumption contains the seeds of disease—

Do not swallow your expectorations. This habit may lead to consumption of the bowels.

Do not spit about the floor, nor into any utensil unless it contains a disinfectant.

Do not spit about the streets.

Indoors, use a special spitting cup or other vessel in which is some disinfectant.

Outdoors, use a pocket handkerchief, or else use a piece of rag or paper, which must be burued as soon as you get home. You can get some disinfectant from the dispenser for use in the spittoon or spitting cup.

The contents of this spittoon should be mixed with some more disinfectant before being emptied.

The spittoon should be emptied into the pan of the w.c. or into a bright fire, but *never* anywhere else, not even into the dust heap.

If you spit into a pocket handkerchief, it must be boiled for five minutes as soon as it is done with.

Keep your room well aired, throw the window wide open when you leave the room, and always keep it open a little at the top all night.

If there is a fire-place in the room, do not stop up the chimney, but always keep it free for the passage of air.

Keep your room clean, do not allow dust to remain on the floor.

Consumptive patients should sleep alone.

Mothers who are consumptive should not suckle their children.

### The Book World for Women and Nurses.

[We invite Correspondence, Criticism, Enquiries, and Notes on Books likely to interest Women and Nurses. Address, Editor, The Hospital (Nurses' Book World), 428, Strand, W.C.]

Handbook of Invalid Cooking. By Mary Bolland. (The Century Company.)

This book, of which we have already given a short preliminary notice, was produced to fill a want for such a work in connection with the Nurse Training School of the Johns Hopkins Hospital at Baltimore, sick cookery being a special feature of the school. The authoress acknowledges her indebtedness to various authorities from whom she has culled in the preparation of her book. Both selections and original matter are judicious and valuable. Part I., which deals with the theory of digestion, contains little that is new, and, therefore, we can pass it by without comment. Part II. is devoted to recipes, many of which are familiar to the English reader, whilst others savour of the land of their origin. Clam broth, cracker gruel, rachahout des arabes, and kumiss are titles of dishes which do not convey much to our understanding; nevertheless, omitting many essentially American items, the selection will be found very complete and useful for the English nurse. No less than 200 pages are devoted to these recipes. We are inclined to think that the distinctive value of this handbook lies within the compass of the last eighty pages, commencing with the diet or menu lists. These menus, if not of necessity strictly adhered to, still offer suggestions for diets in the sick room which will help nurses to vary the patients' meals, a point which is always desirable.

The chapter on serving is really excellent. Miss Bolland commences by observing that as "Cooking is an art, then serving is an art also." It is an art which all nurses should acquire, for patients are always influenced—if only unconsciously—by the manner in which their food is served. In some forms of disease, and with some individuals, life almost depends on the inducement to eat, and in tempting the appetite. A nurse may be very skilful and experienced, and yet not place sufficient value on rendering her patients' meals dainty and appetising. All nurses, and especially those we allude to, will do well to study what Miss Bolland has to say on the subject. She points out that the tray should never be crowded, that everything should be fresh, simple, and clean. A few flowers are a welcome addition. Further, she says, "Little attentions in the way of ornamentation and thoughtfulness as to an invalid's meal are deeply appreciated. They show that an effort has been made to please, and to many sick ones the feeling that they are a constant care to those about them is a very oppressive one. It should be the pleasure of a good nurse to dispel such thoughts. It is the duty of every nurse to do so." Many hints contained in this chapter would be equally beneficially carried out in domestic as well as sick cookery. Following this chapter are twenty pages devoted to the feeding of children. They are well arranged, and carefully considered diets from the first week to the twelfth month are given for use where it is necessary to bring up the child by hand. The chapter on district nursing contains some useful suggestions and advice. A list of cheap dishes are given, also instructions in sweeping, dish cleaning, and fire making,

### THE BOOK WORLD FOR WOMEN AND NURSES—*continued.*

points most essential in a district nurse's training, but which are too often left to the intuitive knowledge of the individual. Throughout the book it is obvious that Miss Bolland thoroughly understands her subject, and has the power of imparting her knowledge to others.

### COMING PUBLICATIONS.

Philanthropic Work of British Women. By the Baroness Burdett-Coutts. (Sampson, Marston, Low, and Co.)

The Baroness Burdett-Coutts, at the request of the President of the Ladies' Committee of the British Commission, H.R.H. Princess Christian, last year became the president of the "Section" charged with the duty of preparing for the Chicago Exhibition a report on the philanthropic work of British women. The Baroness's first object was to collect authentic information about all philanthropic work originated or carried on by English women. This resulted in a collection of reports forming five large volumes, which at the close of the Exhibition will be deposited in the free library in that city. The Baroness has reduced these reports into a readable form, so that it could be presented to the Exhibition in a printed and published volume. The result has been the production of a series of valuable papers covering nearly the whole field of the philanthropic work of women in Great Britain, the colonies, and other parts of the world. These form a volume of unique interest, as no such comprehensive record of charitable endeavour has hitherto been attempted. H.R.H. Princess Christian writes a paper upon the work done by the Royal School of Art Needlework. In addition to a preface, the Baroness contributes two congress papers, and analytical notes on the whole of the original reports. The book will also contain articles by Miss Hesba Stretton and Mrs. Molesworth on "Women's Work for Children"; by Miss Sellers and the Hon. Maude Stanley upon movements for the benefit of girls; by Mrs. Sumner on the "Responsibility of Mothers"; Miss Brooke-Hunt; the Countess Compton; the Hon. Mrs. Stuart Wortley; Miss Marsh and Mrs. C. Garnett on "Navvy Mission Work"; Mrs. Boyd Carpenter; Miss Emily Janes; and Miss Mary Steer are contributors. The authoress of "The Schonberg-Cotta Family" writes upon "Homes of Peace for the Dying"; Miss Florence Nightingale, Lady Victoria Lambton, and Mrs. Malleson upon "Nursing"; "Rosa Mulholland" upon the "Philanthropic Work of Women in Ireland"; and Miss Louisa Twining upon subjects connected with the Poor Law. Mrs. Cashel Hoey gives a general account of the "Philanthropic Work of Women in the British Colonies and the East," which, especially in the case of the Australian colonies, presents many remarkable and interesting features. The other papers in the volume will be found of no less value. It may interest our readers to add that the Baroness has devoted nearly a year's work to the fulfilment of this object

